# GagCM9-Specific CD8^+^ T Cells Expressing Limited Public TCR Clonotypes Do Not Suppress SIV Replication *In Vivo*

**DOI:** 10.1371/journal.pone.0023515

**Published:** 2011-08-26

**Authors:** Lara Vojnov, Mauricio A. Martins, Jorge R. Almeida, Zachary Ende, Eva G. Rakasz, Matthew R. Reynolds, Enrique J. Leon, Kim L. Weisgrau, Benjamin J. Burwitz, Joy M. Folkvord, Marlon G. Veloso de Santana, Patrícia C. Costa Neves, Elizabeth Connick, Pamela J. Skinner, Emma Gostick, David H. O'Connor, Nancy A. Wilson, Myrna C. Bonaldo, Ricardo Galler, David A. Price, Danny C. Douek, David I. Watkins

**Affiliations:** 1 Department of Pathology and Laboratory Medicine, University of Wisconsin-Madison, Madison, Wisconsin, United States of America; 2 Human Immunology Section, Vaccine Research Center, National Institute of Allergy and Infectious Diseases, National Institutes of Health, Bethesda, Maryland, United States of America; 3 Wisconsin National Primate Research Center, Madison, Wisconsin, United States of America; 4 University of Colorado Denver School of Medicine, Denver, Colorado, United States of America; 5 Laboratorio de Biologia Molecular de Flavivírus, Instituto Oswaldo Cruz-FIOCRUZ, Rio de Janeiro, Brazil; 6 Department of Veterinary and Biomedical Sciences, University of Minnesota, St. Paul, Minnesota, United States of America; 7 Instituto de Tecnologia em Imunobiologicos, Fundação Oswaldo Cruz, Rio de Janeiro, Brazil; 8 Department of Infection, Immunity and Biochemistry, Cardiff University, Wales, United Kingdom; Mayo Clinic, United States of America

## Abstract

Several lines of evidence suggest that HIV/SIV-specific CD8^+^ T cells play a critical role in the control of viral replication. Recently we observed high levels of viremia in Indian rhesus macaques vaccinated with a segment of SIVmac239 Gag (Gag_45–269_) that were subsequently infected with SIVsmE660. These seven Mamu-A*01^+^ animals developed CD8^+^ T cell responses against an immunodominant epitope in Gag, GagCM9, yet failed to control virus replication. We carried out a series of immunological and virological assays to understand why these Gag-specific CD8^+^ T cells could not control virus replication *in vivo*. GagCM9-specific CD8^+^ T cells from all of the animals were multifunctional and were found in the colonic mucosa. Additionally, GagCM9-specific CD8^+^ T cells accessed B cell follicles, the primary residence of SIV-infected cells in lymph nodes, with effector to target ratios between 20–250 GagCM9-specific CD8^+^ T cells per SIV-producing cell. Interestingly, vaccinated animals had few public TCR clonotypes within the GagCM9-specific CD8^+^ T cell population pre- and post-infection. The number of public TCR clonotypes expressed by GagCM9-specific CD8^+^ T cells post-infection significantly inversely correlated with chronic phase viral load. It is possible that these seven animals failed to control viral replication because of the narrow TCR repertoire expressed by the GagCM9-specific CD8^+^ T cell population elicited by vaccination and infection.

## Introduction

Human immunodeficiency virus (HIV)- and simian immunodeficiency virus (SIV)-specific CD8^+^ T cells appear to play an essential role in reducing peak and chronic phase HIV/SIV replication. The emergence and expansion of HIV/SIV-specific CD8^+^ T cells coincide with peak viral decline [1]–[4]. Depletion of CD8^+^ cells in both progressor and elite controller (ECs) rhesus macaques leads to increased viral replication in most animals [5]–[8], and HIV/SIV-specific CD8^+^ T cell pressure selects for viral escape [9]–[17]. Select MHC class I alleles, HLA-B*27 and –B*57 in humans and Mamu-B*08 and –B*17 in rhesus macaques, are associated with control of viral replication *in vivo* [13], [18]–[22].

Recent studies suggest that Gag-specific CD8^+^ T cells play a role in reducing viral replication [23]–[30]. Some Gag-specific CD8^+^ T cell clones can recognize and suppress viral replication in SIV-infected CD4^+^ T cells *in vitro* early in the viral life cycle and prior to viral integration [29]. In contrast, Env-, Tat-, and Nef-specific CD8^+^ T cell clones failed to recognize SIV-infected CD4^+^ T cells before twelve hours post-infection when SIV begins to downregulate surface CD4 and MHC class I molecules [29], [31]. Additionally, analysis of approximately 600 HIV-infected patients in Durban, South Africa demonstrated a significant inverse correlation between the breadth of Gag-specific CD8^+^ T cell responses and viral load [26]. Individuals making two or more Gag-specific CD8^+^ T cell responses had significantly lower viral loads than those making one or no Gag-specific CD8^+^ T cell responses. This same study also showed that individuals making one or more Env-specific CD8^+^ T cell response(s) had higher viral loads than those making no Env-specific CD8^+^ T cell responses. Though these are key observations from vaccine- and antiretroviral-naïve HIV patients, the success of vaccine-elicited Gag-specific CD8^+^ T cell responses to lower viral loads *in vivo* is less clear. MHC class I Mamu-A*01^+^ rhesus macaques vaccinated with Gag alone or in combination with Tat, Rev, and Nef only temporally controlled viral replication of SIVmac239 infection [23], [30]. Animals vaccinated with Gag alone made high frequency Mamu-A*01-restricted Gag_181–189_CM9-specific (GagCM9-specific) CD8^+^ T cell responses post-infection and had low viral loads until eighty days post-infection when they began to lose control of viral replication [23]. Additionally, after DNA/Ad5 vaccination with Gag, Tat, Rev, and Nef, followed by SIVmac239 challenge, Mamu-A*01^+^ animals again made high frequency GagCM9-specific CD8^+^ T cell responses [30]. However, there were no significant correlations between peak and chronic phase viremia and the magnitude of Gag- and Nef-specific responses.

The quality of HIV/SIV-specific CD8^+^ T cells may be critical for a successful anti-HIV/SIV immune response. It has been previously shown that ECs have HIV-specific CD8^+^ T cells capable of multiple functions: degranulation and secretion of one or more cytokines [32]. Progressors, on the other hand, have a more limited functional repertoire. Additionally, HIV-specific CD8^+^ T cells from ECs demonstrate superior proliferative capacity as well as increased cytotoxicity of HIV-infected CD4^+^ T cells than HIV-specific CD8^+^ T cells from progressors [33]–[35]. These studies suggest that eliciting multifunctional HIV/SIV-specific CD8^+^ T cells may be critical for a successful vaccine.

The number of public T cell receptor (TCR) clonotypes within a CD8^+^ T cell population may be inversely correlated with viral load [36]. Public clonotypes are defined as epitope-specific TCRβ-chain amino acid sequences that occur in more than one animal. Thus, these are shared T cell receptors that represent an extreme bias in TCR usage. Price *et al.* analyzed the frequency of public TCR clonotypes in the GagCM9-specific CD8^+^ T cell population in vaccinated and unvaccinated SIVmac239-infected rhesus macaques [36]. Vaccinated macaques with low setpoint viral loads had several public TCR clonotypes within the GagCM9-specific CD8^+^ T cell population after vaccination and infection. Additionally, this group observed a significantly inverse relationship between the frequency of public TCR clonotypes elicited by the GagCM9-specific CD8^+^ T cell population and chronic phase viral load in both vaccinated and unvaccinated animals.

In a previous study, we vaccinated seven Mamu-A*01^+^ animals with recombinant Yellow Fever-17D virus containing a portion of SIVmac239 Gag (rYF-17D/SIVGag_45–269_) [37]. Animals were then mucosally challenged between two and four times until infection with low-dose heterologous SIVsmE660. Although all animals made high frequency GagCM9-specific CD8^+^ T cell responses, only one animal successfully controlled viral replication. Our present study, therefore, sought to determine why these high frequency GagCM9-specific CD8^+^ T cells failed to control virus replication. GagCM9-specific CD8^+^ T cells from all animals appeared to be multifunctional and accessed the primary sites of SIV infection; however, GagCM9-specific CD8^+^ T cells from all animals expressed a limited public TCR repertoire after vaccination and infection. In fact, the chronic phase viral load could be predicted by the breadth of public TCR usage in the GagCM9-specific CD8^+^ T cell population.

## Materials and Methods

### Animals and vaccination

Indian rhesus macaques (*Macaca mulatta*) from the Wisconsin National Primate Research Center were cared for according to the regulations and guidelines of the University of Wisconsin Institutional Animal Care and Use Committee, Animal Welfare Assurance No. A3368-01. Full details of the study (UW-Madison Animal Care and Use Protocol No. G00578) were approved by the University of Wisconsin Institutional Animal Care and Use Committee in accordance with the recommendations of the Weatherall report. Once infected, animals were singly housed to prevent cross contamination of SIV infection and spread of opportunistic infections. Animals were closely monitored daily for pain or discomfort and treated accordingly by a veterinarian to ameliorate any suffering. Following progression to AIDS, animals were humanely euthanized. Animals were typed for MHC class I alleles Mamu-A*01, Mamu-A*02, Mamu-B*08 and Mamu-B*17 by sequence-specific PCR [20], [38]. We engineered amino acids 45 to 269 of SIVmac239 Gag into YF17D (rYF17D/SIVGag_45–269_). This was done as previously described by inserting a yellow fever codon-optimized sequence between the genes encoding the viral proteins E and NS1 of yellow fever [37]. All seven animals were subcutaneously given either one or two doses of between 2.3×10^4^ and 2.3×10^5^ plaque forming units (PFU) of rYF17D/SIVGag_45–269_. Additionally one animal, r01056, was primed with 2×10^5^ colony forming units (CFU) of rBCG intradermally containing Gag, Nef, Rev, Tat, and Vif and a second time with 10^7^ CFU of oral rBCG containing Gag, Nef, Rev, Tat ,and Vif.

### Virus stocks, SIV challenge and viral sequencing

SIVmac239, GenBank accession no. M33262, was generated as previously described [39]. Briefly, Vero cells (acquired from ATCC; no. CCL-81) were transfected with plasmid DNA encoding the SIV proviral sequences. One day after transfection, CEMx174 cells (acquired from ATCC; no. CRL-1991) were added to the Vero cultures. Virus was expanded on CEMx174 cells and cell-free supernatant collected two days after peak syncytia formation. SIVsmE660 was generated in a similar manner to SIVmac239, but expanded on fresh peripheral blood mononuclear cells (PBMC) isolated from naïve Indian rhesus macaques. Harvested virus was analyzed by Gag p27 enzyme-linked immunosorbent assay (ZeptoMetrix Corporation) and quantitative RT-PCR prior to animal infection and use in *ex vivo* studies. Approximately eight weeks post-rYF17D/SIVGag_45–269_ boost or mock vaccination, animals were challenged intrarectally with 6×10^6^ viral particles of SIVsmE660 (225 50% tissue culture infective dose [TCID50]). Animals were challenged with this dose and virus every week until infected. Animals were considered SIV positive after a positive viral load determination and were no longer challenged. Animals became infected after between 2–4 virus challenges. Bulk viral Sanger sequencing was performed as previously described [40]. Briefly, vRNA was extracted from plasma using a Qiagen MinElute kit (Valencia, CA). We used a Qiagen One Step RT-PCR kit to amplify the region of Gag containing GagCM9 and its known compensatory mutations. The RT-PCR conditions for this amplicon was: 50°C for 30 min; 95°C for 15 min; 45 cycles of 94°C for 30 s, 53°C for 1 min, and 72°C for 150 s; and 68°C for 20 min. The amplified cDNA was purified using a Qiagen PCR purification kit. A 3730 DNA analyzer (Applied Biosystems) sequenced the cDNA and sequences were assembled using CodonCode Aligner (CodonCode). Pyrosequencing was performed similarly to previously described methods [41]. Briefly, vRNA was extracted by the same method used for bulk viral Sanger sequencing. Viral RNA was reverse transcribed and amplified using the SuperScript III One-Step RT-PCR System with Platinum Taq High Fidelity (Invitrogen, Carlsbad, CA) and MID-tagged primers (454-Life Sciences, Branford, CT) spanning Gag amino acid residues 155–253. The reverse transcription-PCR conditions were as follows: 50°C for 15 min; 94°C for 2 min; 40 cycles of 94°C for 15 sec, 58°C for 30 sec, and 68°C for 50 sec; and 68°C for 5 min. Following PCR clean-up and DNA quantification, amplicons were pooled at equimolar ratios and the final library was diluted to 1.5×10^6^ copies/l. Emulsion PCR was performed at 1.5 copies/bead using the GS Junior Titanium emPCR kit (Lib-A) per manufacturers instructions (Roche, Indianapolis, IN). 5×10^5^ enriched DNA beads were sequenced on a Roche/454 GS Junior.

### Viral load analysis

Viral RNA was extracted using guanidine hydrochloride as previously described [39], [42] for EDTA-anticoagulated plasma and by M48 Virus Mini Kit (Qiagen) for virus production assay samples. Viral RNA was quantified from both plasma and virus production assay supernatant with forward primer (SIV1552), 5′-GTCTGCGTCATCTGGTGCATTC-3′, reverse primer (SIV1635), 5′-CACTAGCTGTCTCTGCACTATGTGTTTTG-3′, and probe, 5′-6-carboxyfluorescein-CTTCCTCAGTGTGTTTCACTTTCTCTTCTGCG-6-carboxytetramethylrhodamine-3′ using the SuperScript III Platinum One-Step Quantitative RT-PCR Kit (Invitrogen) with the LightCycler 1.2 or 480 (Roche) as previously described [39].

### Lymphocyte isolation from gut pinch biopsies

Pinch biopsies were performed as previously described [43]. Briefly, six to eight pinch biopsies of approximately two cubic mm were collected from the jejunem of rhesus macaques by Fujinon FG-100PE pediatric gastroscope. The biopsies were incubated three times in an orbital shaker at 37°C for 30 minutes in complete RPMI (RPMI 1640 supplemented with 15% fetal bovine serum, 2 mM L-glutamine, and 50 µg/ml antimycotic/antibiotic- all purchased from HyClone Laboratories, Inc.) containing 15 µg of collagenase type II (Sigma)/ml. The supernatant containing lymphocytes was collected after each incubation period, washed twice, and pooled. Cells were filtered using a nylon mesh to remove large debris. Lymphocytes were isolated by overlaying the cells on an isotonic Percoll (Amersham-Pharmacia) gradient (40% layered over 90%) and centrifuging for 30 minutes at 800 rpm. Lymphocytes were collected from the 90%/40% interface using a transfer pipette and washed with complete RPMI.

### *In situ* lymph node staining

*In situ* tetramer staining combined with immunohistochemistry was performed as described previously [44]. Biotinylated Mamu-A*01 molecules loaded with GagCM9 peptide were purchased from Immunomics while irrelevant negative control (FLPSDYFPSV) peptide was obtained from the National Institute of Allergy and Infectious Diseases. Fresh tissues embedded in low melt agarose were cut into 200 micron thick sections and incubated with MHC tetramers at a concentration of 0.5 ug/ml, mouse anti-CD20 antibodies (Novacastra) diluted 1∶200, and rat-anti-human CD8 antibodies (AbD Serotec) diluted 1∶200 in 1 ml of cold phosphate buffered saline containing 100 mg/ml heparin (PBS-H) with 2% normal goat serum at 4°C overnight. Sections were then washed with chilled PBS-H, fixed with 4% paraformaldehyde for 2 h at room temperature, and again washed with PBS-H. Sections were then incubated with rabbit anti-FITC antibodies (BioDesign) diluted 1∶10,000 in blocking solution, and incubated at 4°C on a rocking platform overnight. Sections were washed with PBS-H and incubated with Cy3-conjugated goat anti-rabbit antibodies (Jackson ImmunoResearch) diluted 1∶5000, Cy5-conjugated anti-rat antibodies (Jackson ImmunoResearch) diluted 1∶2000, and Alexa 488-conjugated goat anti-mouse antibodies (Molecular Probes) diluted 1∶2000 in blocking solution for 1 to 3 days, washed again, post-fixed with 4% paraformaldehyde for 1 hour, and then mounted on slides with warmed glycerol gelatin (Sigma) containing 4 mg/ml *n*-propyl gallate. Stained sections were imaged using a Fluoview 1000 microscope (Olympus). To detect SIV RNA-producing cells, four 6 µm tissue sections (each section approximately 30 microns apart) of snap frozen lymph node were stained using a riboprobe for full length SIVmac239 as previously described to detect HIV RNA-producing cells in humans [45]. All sections were counterstained with CD20 antibody to define follicular regions. All sections were visualized and positive cells counted and classified as inside or outside of the follicle. Area for the follicular region, extrafollicular region and total tissue was determined using Qwin Pro on a Leica DMR Fluorescent Microscope (Leica). Data were reported as SIV^+^ cells per mm^2^ and replicates were combined.

### Quantification of tetramer-binding cells *in situ*

Three-dimensional montage images were created from lymph node sections stained with tetramers, anti-CD8 antibodies and anti-CD20 antibodies using a FluoView 1000 confocal microscope with a 20× objective lens and 3 µm z-steps as previously described [44]. Confocal z-series were collected from approximately 5 µm from surface of staining to as deep as the CD20 staining penetrated, approximately 35 µm into the tissue. Each montage consisted of several 800×800 pixel fields stitched together using Olympus Fluoview FV1000 software. Fluoview FV1000 software was also used to delineate follicular and extrafollicular areas and to count tetramer-binding cells in delineated areas. Follicular areas were identified using CD20 staining. To prevent bias, the red tetramer channel was turned off when follicular and extrafollicular areas were delineated. Areas that showed loosely aggregated B cells that were ambiguous as to whether the area was a follicle were not scored. Cell counts were done on single z-scans. While doing the cells counts, we stepped up and down through the z-scans to distinguish tops and bottoms of cells from non-specific background staining. We also stepped up and down through the z-scans to evaluate the relative abundance and localization of tetramer positive cells inside of follicles. Image J software was then used to trace and measure the areas delineated for cell counts. Cell counts were performed on both Mamu A*01-restricted GagCM9 and negative control Mamu A*01-restricted FLP stained sections.

### Immunological assays

Tetramer staining was performed as previously described [30]. Briefly, 5×10^5^ to 1×10^6^ fresh, unstimulated PBMC from rhesus macaques were stained for 1 hour at 37°C with Mamu-A*01-restricted GagCM9 tetramer labeled with PE or APC (5 µg/ml). Cells were stained with CD3 FITC (BD Biosciences, clone SP34-2) and CD8 PerCP (BD Biosciences, clone 53-6.7) for an additional 30 minutes. The cells were then washed twice with FACS buffer and fixed with 1% paraformaldehyde. Sample data were acquired using CellQuest Pro software on a SORP BD LSR II equipped with a 50 mW 405 violet, a 100 mW 488 blue, and a 50 mW 640 red laser and were analyzed by FlowJo 9.1 (TreeStar, Inc.). IFN-γ Enzyme-linked immunospot (ELISPOT) (Mabtech) assays were performed using freshly isolated PBMC from rhesus macaques as previously described [46]. Briefly, 1×10^5^ PBMC per well were stimulated with peptide in a precoated ELISPOT plate according to the manufacturer's instructions and incubated at 37°C in a 5% CO_2_ incubator overnight. All tests were performed in duplicate using individual peptides at 10 µM or peptide pools (10 15-mer peptides overlapping by 11 amino acids spanning the entire SIV genome) at 1 µM. Fifteen-mer peptides were provided by the NIH AIDS Research and Reference Reagent Program (Germantown, MD). Plates were imaged using an ELISPOT reader (Autoimmun-Diagnostika), counted by ELISPOT Reader version 4.0 (Autoimmun-Diagnostika), and analyzed as previously described [30]. Multifunctional intracellular cytokine staining was performed as previously described [30], [32], [47]. Briefly, freshly isolated PBMC from rhesus macaques were incubated with 5 µM peptide, anti-CD28 (clone L293; BD Biosciences), anti-CD49d (clone 9g; Pharmigen), and CD107a PE (clone H4A3; BD Biosciences) antibodies and 5 µg per test of Brefeldin A and Golgi Stop overnight at 37°C in a 5% CO_2_ incubator. Cells were stained for the surface expression of CD3 PerCP-Cy5.5 (clone SP34; BD Biosciences), CD8 APC-Cy7 (clone SK1; BD Biosciences), CD14 PE-Texas Red (clone RMO52; Beckman Coulter), and CD19 PE-Texas Red (clone JA.119; Beckman Coulter), washed twice with FACS buffer, and fixed with 1% paraformaldehyde. Cells were then permeabilized with 0.1% Saponin buffer, intracellularly stained with IFN-γ PE-Cy7 (clone 4S.B3; BD Biosciences), TNF-α Alexa700 (clone MAb11; BD Biosciences), IL-2 APC (clone MQ1-17H12; BD Biosciences), and MIP-1β FITC (clone 24006; R&D Systems), washed twice with Saponin buffer, and fixed with 1% paraformaldehyde. Sample data were acquired using CellQuest Pro software on a SORP BD LSR II equipped with a 50 mW 405 violet, a 100 mW 488 blue, and a 50 mW 640 red laser and were analyzed by FlowJo 9.1 (TreeStar, Inc.). Analysis and presentation of distributions were performed using Pestle and SPICE version 5.1, downloaded from http://exon.niaid.nih.gov/spice [48].

### T cell receptor sequencing

T cell receptors were amplified and sequenced as previously described [36]. GagCM9-specific tetramer-labeled CD8^+^ T cells were sorted viably from frozen PBMC from rhesus macaques into 1.5 mL microtubes containing 150 µL RNAlater (Applied Biosystems). The median number of sorted cells was 400 cells for the post vaccination time point and 10,000 for the post infection time point. All expressed TCRβ gene products were amplified without bias using an anchored template-switch RT-PCR (SMARTer RACE Clontech); amplicons were subcloned in the pGEM-T easy vector system (Promega) and cloned by transformation of competent DH5α E. coli (Invitrogen). Selected colonies were amplified by PCR with standard M13 primers and sequenced using the Sanger technique. A minimum of 50 clones were generated and analyzed per sample.

## Results

### Immunodominant, multifunctional Mamu-A*01-restricted GagCM9-specific CD8^+^ T cells do not control viral replication

**Figure 1:**
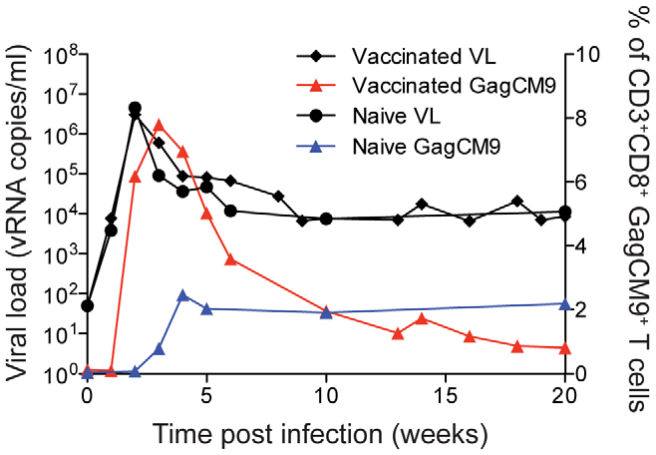
Viral load and GagCM9-specific CD8^+^ T cell tetramer staining. The group mean viral load of the vaccinated animals (black diamonds and connecting lines) was comparable to the two mock-vaccinated, SIVsmE660-infected control animals (black circles and connecting lines); however, the vaccinated animals had higher frequencies of GagCM9-specific CD8^+^ T cells (red triangles and connecting line) than the mock-vaccinated control animals (blue triangles and connecting line).

We vaccinated seven Mamu-A*01^+^ Indian rhesus macaques with Yellow Fever-17D expressing amino acids 45 to 269 of the SIVmac239 Gag protein (rYF-17D/SIVGag_45–269_) (Table 1). The SIV transgene was inserted between the YF-17D Envelope and NS1 genes as detailed previously [37]. Approximately two months after vaccination, animals were repeatedly challenged with six million viral copies of the swarm SIVsmE660 virus. It took between two to four challenges to infect all seven animals. These animals had an average peak of viral replication at two weeks post infection of three million viral RNA copies/ml. By thirty-two weeks post-infection, six of the seven animals had a viral load of greater than 8,000 viral RNA copies/ml (Figure S1a–g). The average viral load at thirty-two weeks post-infection was >60,000 viral RNA copies/ml (Figure 1). Only animal r02049 maintained a viral load below 1,000 viral RNA copies/ml throughout the chronic phase of infection.

**Table 1:** 

Animal	Gender	Prime	Boost	# of challenges	MHC class I alleles	MHC class II alleles	GagCM9 CD8^+^ T cell avidity (nM)
r01056	M	2.0×105 CFU rBCG i.d., 107 CFU rBCG oral of GNRTV	2.0×105 PFU rYF17D/SIVGag45–269	3	A*01, B*17, B*29, B*52	DRB1*0401	32
r02013	F	none	2.0×105 PFU rYF17D/SIVGag45–269	4	A*01, B*22	none tested	19
r02042	F	2.3×105 PFU rYF17D/SIVGag45–269	2.0×105 PFU rYF17D/SIVGag45–269	2	A*01, B*22, B*29, B*30	DPB1*06	7
r02049	M	2.3×105 PFU rYF17D/SIVGag45–269	2.0×105 PFU rYF17D/SIVGag45–269	2	A*01, B*46	DRBw*201, DPB1*06, DRB1*0303	44
r03130	M	2.3×104 PFU rYF17D/SIVGag45–269	2.0×105 PFU rYF17D/SIVGag45–269	2	A*01, B*29, B*46	DRB*w201, DPB1*06	36
r04051	F	none	2.0×105 PFU rYF17D/SIVGag45–269	2	A*01, A*08, B*30, B*46	DRB1*0401, DRB1*0306, DRB1*1003	73
r04091	M	2.3×104 PFU rYF17D/SIVGag45–269	2.0×105 PFU rYF17D/SIVGag45–269	4	A*01, B*22, B*30, B*46	DRB*w201, DPB1*06, DRB1*0303	38

An additional two Mamu-A*01^+^ animals, r02109 and r02110, were vaccinated with empty rYF-17D and subsequently challenged with SIVsmE660. Neither animal controlled viral replication under 2,000 viral RNA copies/ml in the chronic phase of infection (Figure S1h and i). Six of the seven vaccinees had viral loads comparable to, if not higher, than the two control animals. The group mean viral load of the vaccinees was similar to the viral loads of the mock-vaccinated controls (Figure 1), illustrating that the vaccinated animals failed to control viral replication *in vivo*.

Throughout infection, we measured the Mamu-A*01-restricted GagCM9-specific CD8^+^ T cell response from all animals using MHC class I tetramers (Figure S1a–i). Six of the seven vaccinated animals had a peak of the GagCM9-specific CD8^+^ T cell response at week two or three post-infection that ranged between 2.72–38.6% of total CD3^+^CD8^+^ T cells. Both of the mock-vaccinated controls, r02109 and r02110, also made GagCM9-specific CD8^+^ T cell responses post-infection. Animal r02109 had a peak GagCM9-specific CD8^+^ T cell response at week six post-infection of just over 9% of total CD3^+^CD8^+^ T cells (Figure S1h). Animal r02110, however, maintained its GagCM9-specific CD8^+^ T cell frequency below 1% of total CD3^+^CD8^+^ T cells throughout infection (Figure S1i). Overall, vaccinated animals had 3.2 fold higher peak frequencies of GagCM9-specific CD8^+^ T cells than the control animals (Figure 1). Additionally, the GagCM9-specific CD8^+^ T cells from all animals displayed a high avidity profile indicating the GagCM9-specific CD8^+^ T cells required few MHC-epitope complexes on the surface of a target cell to induce cytokine secretion (Table 1). Despite the rapidly induced high frequency anamnestic GagCM9-specific CD8^+^ T cells in the vaccinated animals, viral replication in six of the seven vaccinated animals was comparable to the mock-vaccinated controls. Thus, it appeared the high frequency of the vaccine-induced GagCM9-specific CD8^+^ T cells failed to control viral replication *in vivo*.

**Figure 2:**
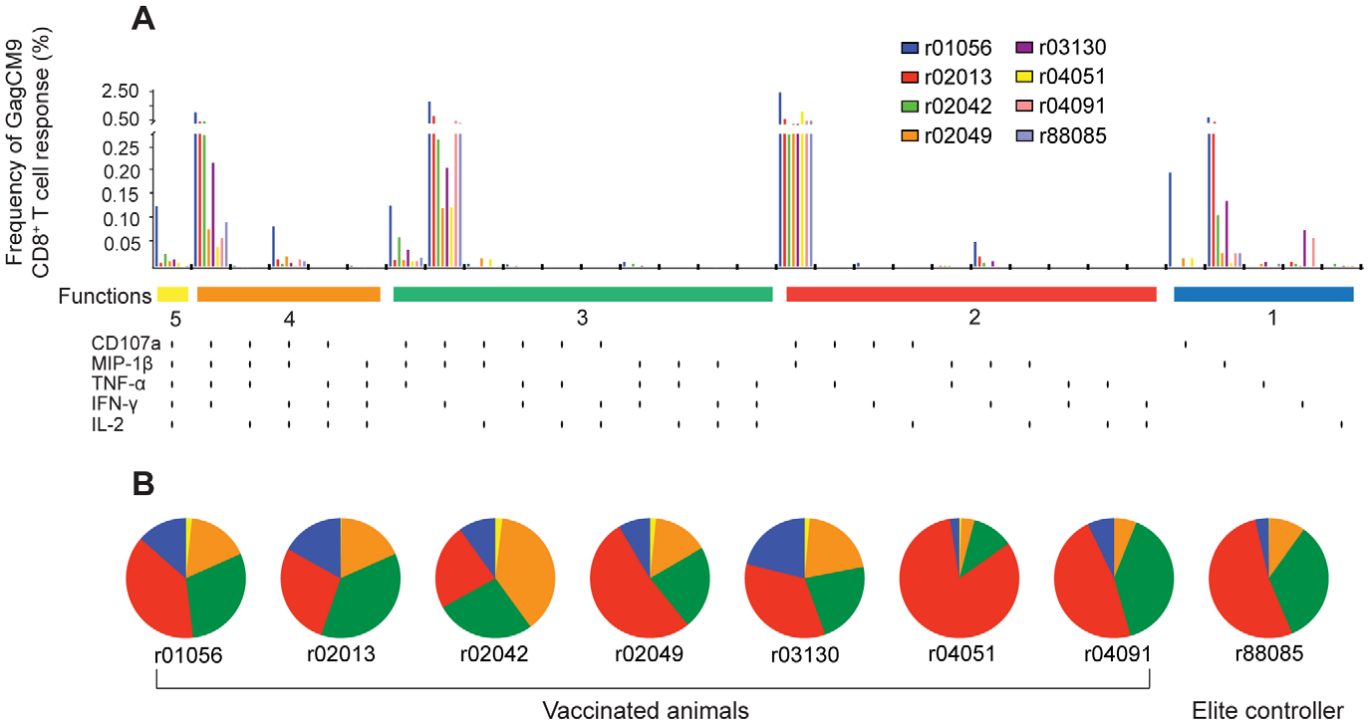
Multifunctional GagCM9-specific CD8^+^ T cell responses. (a, b) Using intracellular cytokine staining, we analyzed the ability of GagCM9-specific peptide-stimulated PBMC to degranulate (CD107a) and secrete MIP-1β, TNF-α, IFN-γ, and/or IL-2 at week ten post-infection. (a) Bar graphs indicate the GagCM9-specific CD8^+^ T cell response frequency for each molecule alone or in combination with the other tested molecules from each animal. Each vertical black line below the horizontal colored bars indicates positivity for CD107a, MIP-1β, TNF-α, IFN-γ and/or IL-2. (b) Pie graphs indicate the percentage of GagCM9-specific CD8^+^ T cells that had five molecules (yellow), four molecules (orange), three molecules (green), two molecules (red) or one molecule (blue). Animal r88085 was a Mamu-A*01^+^ delta-nef-vaccinated, SIVsmE660-infected elite controller from a previous study who mounted a GagCM9-specific CD8^+^ T cell response.

Six out of the seven animals did not control viral replication even with such high frequency GagCM9-specific CD8^+^ T cell responses. We, therefore, sought to determine why these GagCM9-specific CD8^+^ T cells failed to control viral replication. First, we tested the multifunctional capacity of chronic phase GagCM9-specific CD8^+^ T cells from our seven animal cohort using an intracellular cytokine stain assay (ICS) (Figure 2). The multifunctional capacity of HIV-specific CD8^+^ T cells has been associated with elite control of HIV-infected patients [32]. HIV-specific CD8^+^ T cells that degranulate while concurrently secreting multiple cytokines have been correlated with low patient viral loads, while HIV-specific CD8^+^ T cells from progressors consistently failed to perform all five functions and were generally less multifunctional. We hypothesized that the GagCM9-specific CD8^+^ T cells from these animals might not express an appropriate range of cytokines and thus could not control viral replication. We, therefore, compared the cytokine profile of the vaccinated animals at approximately ten weeks post-infection to a Mamu-A*01^+^ delta-nef-vaccinated, SIVsmE660-infected EC, r88085, which is successfully controlling viral replication [49]. Five of the seven vaccinated animals had a small percentage of GagCM9-specific CD8^+^ T cells that performed all five functions tested (Figure 2a and b). This fraction of GagCM9-specific CD8^+^ T cells degranulated as measured by CD107a staining and secreted all four tested molecules: MIP-1β, TNF-α, IFN-γ, and IL-2. Less than 25% of GagCM9-specific CD8^+^ T cells performed only one function tested. Though slight differences were seen, six of the seven vaccinated animals had a high percentage of GagCM9-specific CD8^+^ T cells that performed three or more measured functions. Most of the GagCM9-specific CD8^+^ T cell responses from the seven vaccinated animals therefore secreted several cytokines and were also comparable to the multifunctional GagCM9-specific CD8^+^ T cell response of r88085, an EC. However, the GagCM9-specific CD8^+^ T cells from the vaccinated animals did not control viral replication.

### GagCM9-specific CD8^+^ T cells access the gut mucosa and B cell follicles in lymph nodes

**Figure 3:**
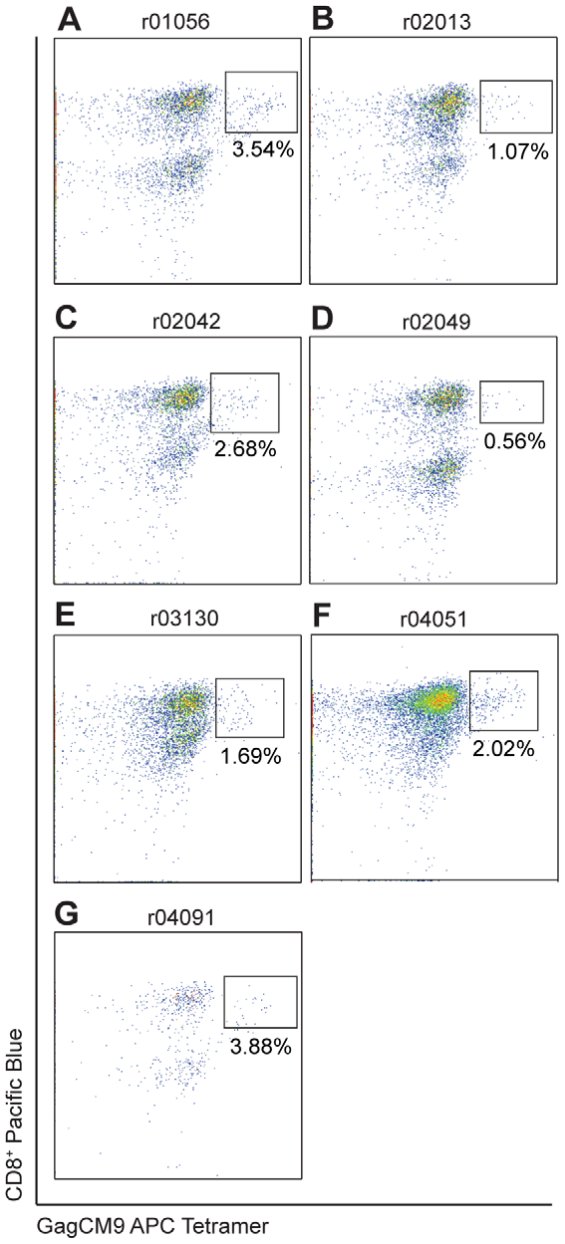
GagCM9-specific CD8^+^ T cells access the gut mucosa. Gut biopsies were performed during the chronic phase of infection for each animal. Harvested gut lymphocytes were stained for GagCM9 APC tetramer, CD3 FITC and CD8 Pacific Blue. Each panel was gated on CD3^+^ lymphocytes.

Because the high frequency, multifunctional GagCM9-specific CD8^+^ T cells did not reduce viral replication *in vivo*, we next speculated that these cells might not be accessing the major sites of infection, which would explain their inability to control viral replication in SIV-producing cells. The gut mucosa is a primary site of HIV and SIV infection [50], [51]. At approximately fifteen weeks post-infection we biopsied the gut of all seven animals and extracted lymphocytes. GagCM9 tetramer staining of the colonic lymphocytes showed that between 0.56%–3.88% of the CD3^+^ lymphocytes were GagCM9-specific CD8^+^ T cells (Figure 3a–g). The presence of these cells in the gut during the chronic phase of infection indicates that although recruited to one known site of infection, they were still not capable of controlling viral replication.

**Figure 4:**
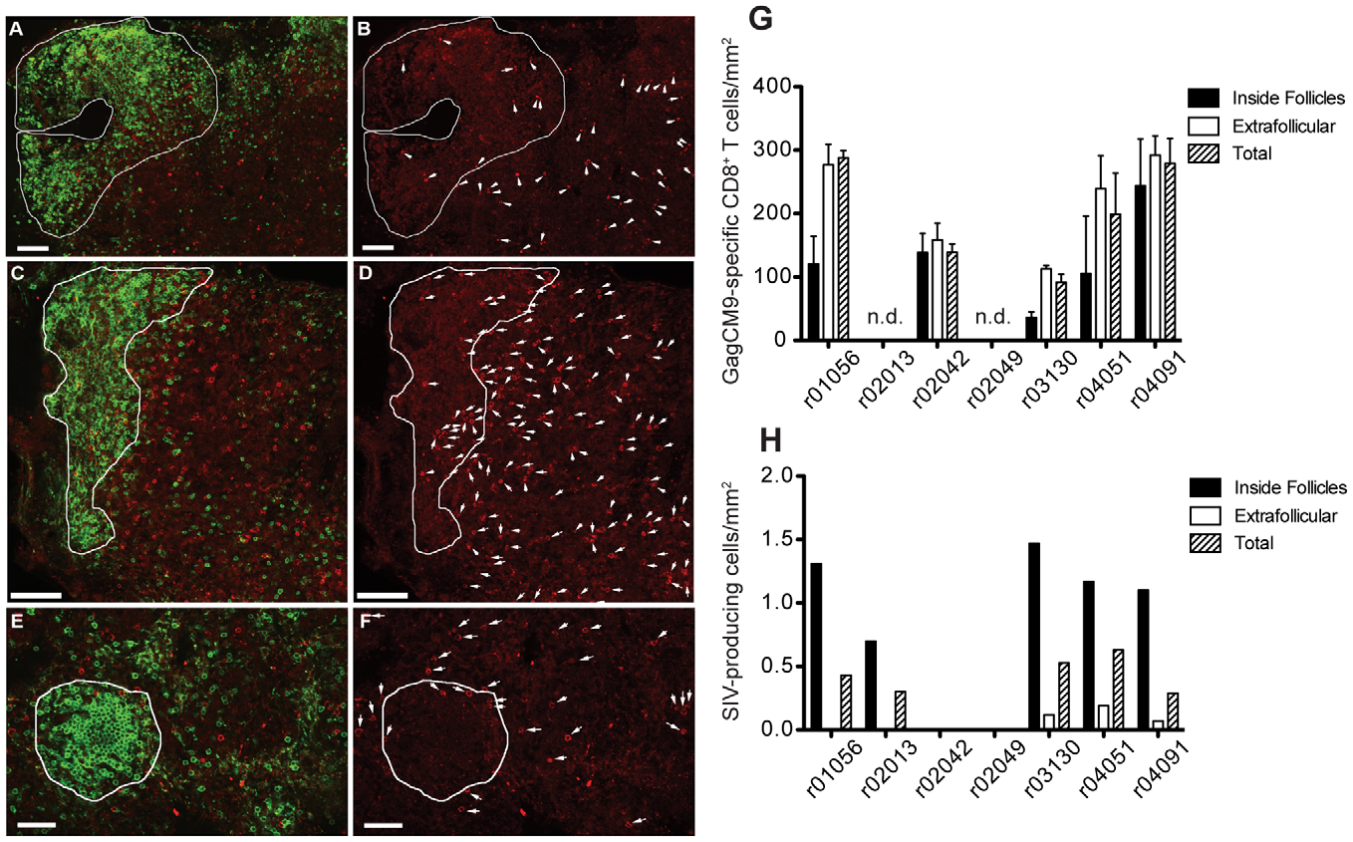
GagCM9-specific CD8^+^ T cells reside in the lymph nodes of infected animals at high effector to target ratios. Representative images of Mamu A*01-restricted GagCM9-specific tetramer positive CD8^+^ T cells (red) and CD20^+^ B cells (green) in lymph node sections taken during the chronic phase of infection from animals r03130 (a, b), r04051 (e, f), and r04091 (c, d). B cell follicles are delineated with a white line. The images to the right (b, d, f) show the same field as presented on the left (a, c, e) with only the red tetramer stain shown. Each tetramer-binding cell is indicated with a white arrow. (g) The frequency of GagCM9-specific tetramer positive CD8^+^ T cells per mm^2^ inside and outside of the B cell follicle as well as total tissue was calculated for each animal. (h) The frequency of SIV producing cells per mm^2^ inside and outside the B cell follicle as well as total tissue was calculated for each animal. Animals r02042 and r02049 did not have any positive cells in the sections examined.

Lymph nodes also have high concentrations of SIV-producing cells; therefore, we next biopsied lymph nodes from the chronic phase of infection to determine the localization and abundance of GagCM9-specific CD8^+^ T cells [52]. *In situ* GagCM9 tetramer staining showed high concentrations of GagCM9-specific CD8^+^ T cells in the lymph nodes of five of the seven animals as well as the presence of GagCM9-specific CD8^+^ T cells in the B cell follicle (Figure 4b, d, f, and g), the primary site of infected cells in the lymph node ([53], Figure 4h). Although concentrated in the extrafollicular regions of the lymph nodes, GagCM9-specific CD8^+^ T cells were found in relatively high concentrations within the B cell follicles (Figure 4g). *In situ* hybridization of the lymph nodes found SIV-producing cells concentrated inside the B cell follicles with very few residing in the extrafollicular region (Figure 4h). Comparing the abundance of GagCM9-specific CD8^+^ T cells with the quantity of SIV-producing cells inside the B cell follicle of the lymph node samples from each animal showed a high effector to target ratio of approximately 20–250 GagCM9-specific CD8^+^ T cells per SIV-producing cell (Figure 4g and h). The effector to target ratio increased further when the total lymph node tissue was examined. The inability of GagCM9-specific CD8^+^ T cells to access sites of infection did not, therefore, explain the high viral loads in these animals.

### Viral escape from the GagCM9-specific CD8^+^ T cell response does not account for the lack of viral control

**Figure 5:**
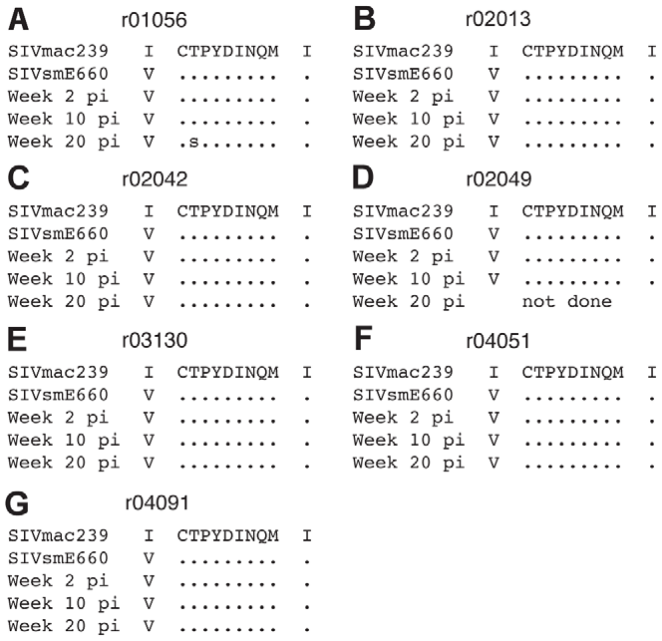
Viral sequencing of Gag containing the GagCM9 CD8 epitope and its known compensatory mutation amino acid locations showed little viral escape. (a–g) We performed bulk viral Sanger sequencing at weeks two, ten, and twenty post-infection of the GagCM9 epitope and surrounding amino acids. The SIVsmE660 stock contains the isoleucine to valine GagCM9 upstream compensatory mutation at position 161, which all animals maintained throughout the acute and chronic phases of infection. Dots indicate no amino acid deviation from SIVmac239. Animal r02049 had no detectable virus at week 20; therefore, its virus could not be sequenced.

We next hypothesized that GagCM9-specific CD8^+^ T cell pressure had selected for viral escape soon after infection. If so, the GagCM9-specific CD8^+^ T cells would no longer be able to recognize SIV-infected cells and therefore fail to control viral replication. However, it has previously been shown that Gag-vaccinated rhesus macaques did not select for viral escape in the GagCM9 epitope until 30 weeks post-SIVsmE660 infection [54]. Additionally, in animals that initially controlled SIVsmE660 infection, the emergence of escape mutations in the GagCM9 epitope correlated with viral breakthrough [54]. To examine the potential role of viral escape from the GagCM9-specific CD8^+^ T cell response, we sequenced plasma from all seven animals at weeks two, ten and 20 post-infection (Figure 5). The consensus sequence of SIVsmE660 contains an isoleucine to valine amino acid change at position 161 at the upstream compensatory GagCM9 mutation, I161V [55]. Besides this change, only virus from animal r01056 contained amino acid variation at 20 weeks post-infection (Figure 5a); a position two mixed base of threonine and serine. The downstream compensatory GagCM9 position 206 isoleucine to valine, I206V, mutation was not seen in any of the animals at any timepoint. Additionally, we performed pyrosequencing of RT-PCR amplicons from each timepoint. At weeks two and ten post-infection, the GagCM9 epitope from all animals contained no amino acid variation (Figure S2). At week 20 post-infection, pyrosequencing confirmed the position two threonine to serine substitution in r01056 with the mutation seen in approximately 50% of the viral population. Less than 25% of the viral population from the other six animals at week 20 post-infection contained an amino acid replacement in the GagCM9 epitope (Figure S2). Viral escape, therefore, does not explain the failure of the GagCM9-specific CD8^+^ T cells to control viral replication during both the acute and early chronic phases of infection.

### Vaccinated animals have few public TCR clonotypes within the GagCM9-specific CD8^+^ T cell population pre- and post-infection

**Figure 6:**
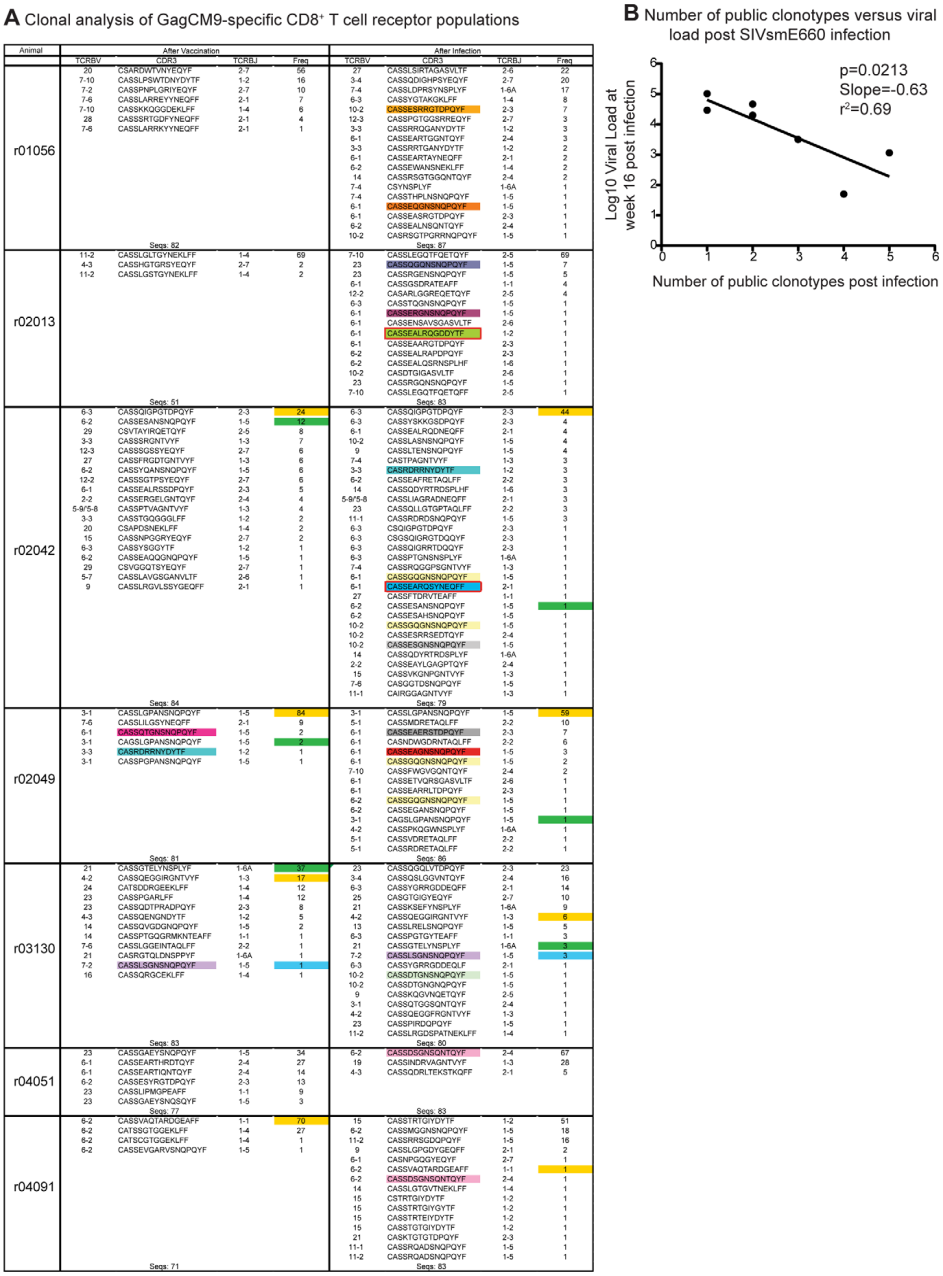
GagCM9-specific CD8^+^ T cell receptor clonal analysis. (a) TCRβ CDR3 sequences, TCRβV and TCRβJ usage, and the relative frequency of TCR clonotypes in the GagCM9-specific CD8^+^ T cell population were analyzed for each animal after vaccination and three weeks after infection. Colored boxes in the CDR3 column indicate public TCR clonotypes (GagCM9-specific TCRβ-chain amino acids that occur in more than one Mamu-A*01^+^ rhesus macaque), while colored boxes in the frequency column indicate TCR clonotypes that were maintained from post-vaccination to post-infection. (b) The week 16 viral load from each animal was correlated with the number of public TCR clonotypes within the GagCM9-specific CD8^+^ T cell population present at week three post infection.

Thus far, we have determined that our cohort of vaccinated animals have high frequency, multifunctional GagCM9-specific CD8^+^ T cells that accessed major sites of SIV infection, but yet were still unable to control viral replication. The number of public TCR clonotypes within the GagCM9-specific CD8^+^ T cell population may be inversely correlated with viral load [36]. Public clonotypes are defined as epitope-specific TCRβ-chain amino acid sequences that occur in more than one animal. Thus, these are shared T cell receptors that represent an extreme bias in TCR usage. We, therefore, hypothesized that our vaccinated cohort would have few public TCR clonotypes within the GagCM9-specific CD8^+^ T cell population post-infection. Post-vaccination and at four weeks post-infection, GagCM9-specific CD8^+^ T cells were sorted from PBMC from all seven vaccinated animals. The TCRs expressed by these cells were then sequenced and analyzed. Interestingly, five out of the seven animals did not have any public clonotypes in the GagCM9-specific CD8^+^ T cell population post-vaccination and pre-infection (Figure 6a). Animal r03130 had only one public clonotype, while r02049, the elite controller animal, had two public clonotypes pre-infection. Post-infection, all animals had GagCM9-specific CD8^+^ T cells that expressed public TCR clonotypes. T cells from these animals expressed between one and five public clonotypes post-infection with GagCM9-specific CD8^+^ T cells from animals r02042 and r02049 expressing five and four public clonotypes post-infection, respectively. Interestingly, GagCM9-specific CD8^+^ T cells from animal r02042 expressed more public clonotypes than r02049, the elite controller animal; however, r02042 failed to control viral replication.

Few of the GagCM9-specific TCR clonotypes expressed post-vaccination were maintained after infection. Only four of the seven animals maintained GagCM9-specific TCR clonotypes after infection. Only two of the eight maintained GagCM9-specific TCR clonotypes in those four animals had frequencies greater than 7% of the total TCR clonotypes post-infection. Additionally, the frequency of only one vaccine-induced, maintained GagCM9-specific TCR clonotype expanded post-infection. A TCR clonotype from r02042 increased from 24% of the total GagCM9-specific TCR population post-vaccination to 44% post-infection. Interestingly, there was a statistically significant inverse correlation between the number of public clonotypes expressed by GagCM9-specific CD8^+^ T cells post-infection and chronic phase viral load (Figure 6b, p = 0.0213). Animals that had three, four, or five public TCR clonotypes specific for GagCM9 post-infection had significantly lower week 16 viral loads than animals with only one or two public TCR clonotypes. Though all animals had at least one public GagCM9-specific TCR clonotype expressed post-infection, our data indicate that the limited TCR repertoire within the GagCM9-specific CD8^+^ T cell response might be a contributing factor for the lack of control of viral replication.

## Discussion

In a previous study [37], we vaccinated seven Mamu-A*01^+^ rhesus macaques with rYF-17D/SIVGag_45–269_. After infection with the swarm SIVsmE660 virus, six of the seven animals failed to control viral replication. No control of acute phase viral replication was observed in any animal, while only r02049 controlled viral replication during the chronic phase of infection. Additionally, there were no differences between the vaccinated group mean viral load and the viral loads of the two mock-vaccinated Mamu-A*01^+^ control animals. Interestingly, however, all vaccinated animals made high frequency, anamnestic GagCM9-specific CD8^+^ T cell responses that dominated the anti-SIV immune response in all animals (Figure S3). More than 7% of the CD3^+^CD8^+^ T cells were specific for the GagCM9 epitope. These GagCM9-specific CD8^+^ T cells were multifunctional, that is greater than 75% of GagCM9-specific CD8^+^ T cells in each animal degranulated and secreted several molecules simultaneously. Additionally, the GagCM9-specific CD8^+^ T cells from all animals accessed the gut mucosa as well as B cell follicles in the lymph node, the primary residences of SIV-producing cells. The number of public TCR clonotypes expressed by the GagCM9-specific CD8^+^ T cell populations of this cohort, however, significantly inversely correlated with chronic phase viral load.

HIV-infected ECs have multifunctional HIV-specific CD8^+^ T cells as compared to HIV-infected progressors [32]. This implies that maintaining HIV/SIV-specific CD8^+^ T cells capable of concomitantly secreting IFN-γ, TNF-α, IL-2, and MIP-1β and upregulating cell surface expression of CD107a may be critical to reducing viral loads *in vivo*. SIVsmE660 infection induced multifunctional GagCM9-specific CD8^+^ T cells comparable to those from a delta-nef-vaccinated, SIVsmE660-infected EC; however, these cells failed to control viral replication. The multifunctional profile of the GagCM9-specific CD8^+^ T cells from the EC in this study, r02049, was very similar to the multifunctional profiles of r01056 and r03130, two animals with high viral loads. Though the ability of HIV/SIV-specific CD8^+^ T cells to secrete several cytotoxic molecules and degranulate simultaneously likely plays a role in reducing viral replication, our study indicates that despite the presence of these types of antigen-specific CD8^+^ T cells, viral replication continued unabated.

A previous study has suggested that SIV-specific CD8^+^ T cells only partially controlled viral replication because they expanded too late after SIVmac239 infection and were low or undetectable in the gut mucosa [56]. Even though the vaccine-induced GagCM9-specific CD8^+^ T cells in our current study expanded to high frequencies by two weeks post-SIVsmE660 infection, the SIV-specific CD8^+^ T cell response may have simply arrived too late to control viral replication and prevent CD4^+^ T cell loss in the gut mucosa. Additionally, while the decline of peak viremia was concomitant with the expansion of GagCM9-specific CD8^+^ T cells, the reduction of peak viremia in these animals may have been due to other factors such as the loss of targets cells, other SIV-specific CD8^+^ T cells, SIV-specific CD4^+^ T cells, innate features, etc.

The inability of GagCM9-specific CD8^+^ T cells to control viral replication *in vivo* may be due to the lack of vaccine- and infection-induced public TCR clonotypes within the GagCM9-specific CD8^+^ T cell population. Public TCR clonotypes expressed by GagCM9-specific CD8^+^ T cells were seen pre-infection in only two vaccinated animals: two public TCR clonotypes in r02049 and one in r03130. An average of 2.5 public TCR clonotypes in the GagCM9-specific CD8^+^ T cell population were elicited after SIVsmE660 infection. In fact, the number of public TCR clonotypes present at week 3 post-infection significantly correlated with the week 16 viral load. A similar earlier study analyzed the frequency of public TCR clonotypes in the GagCM9-specific CD8^+^ T cell population elicited by a DNA/Ad5 prime/boost vaccination regimen encoding SIVmac239 Gag, Tat, Rev, and Nef of Mamu-A*01^+^ SIVmac239-infected macaques [36]. More public TCR clonotypes were elicited in the GagCM9-specific CD8^+^ T cell population after vaccination and after infection than in our current study, particularly in animals that controlled viral replication. ECs in this previous study had an average of four public TCR clonotypes after vaccination and almost seven public TCR clonotypes after infection. The correlation data from the previous study [36] and our current study are surprisingly consistent with similar slopes (−0.55 versus −0.63) and r^2^ values (0.66 versus 0.69) when we compare the number of public TCR clonotypes elicited in the GagCM9-specific CD8^+^ T cell population of vaccinated animals with chronic phase viral load. CD8^+^ T cells with public TCR clonotypes expand quickly upon antigen stimulation and it has been suggested that public TCR clonotypes may have increased recognition of epitope variants compared to private TCR clonotypes [36], [57], [58]. The inability of our vaccinated animals to control viral replication *in vivo* may, therefore, have been due to the paucity of public TCR clonotypes expressed by the GagCM9-specific CD8^+^ T cell population.

It is difficult to understand why such high frequencies of GagCM9-specific CD8^+^ T cells had no effect on viral replication. Animal r01056 had approximately 40% of its CD3^+^CD8^+^ T cells directed against the virus during the acute phase, yet there was no effect on viremia. High concentrations of an effective antiretroviral drug would have resulted in control of viral replication with subsequent escape. Escape from the GagCM9-specific CD8^+^ T cell response did not occur until week 20 post-infection in r01056 and its GagCM9 epitope was intact at weeks two and ten post-infection. It might be argued that the breadth of the CD8^+^ T cell response is important [5], [26], [28], [59], [60] and that 20 different low frequency CD8^+^ T cell responses against several epitopes would have been more effective than a single high frequency CD8^+^ T cell response. This would be understandable if the GagCM9 epitope had escaped early, but this was not the case. One implication of this result could be that some CD8^+^ T cell responses are more efficacious than others. This would, therefore, be epitope-dependent since the GagCM9-specific CD8^+^ T cell responses in this study appeared to be effective by all of our classical measurements. Finally, our data suggest that the GagCM9-specific CD8^+^ T cell popoulations from the rYF-17D/SIVGag_45–269_-vaccinated, SIVsmE660-infected animals failed to reduce viral replication due to the limited public TCR repertoires elicited by the GagCM9-specific CD8^+^ T cell populations post-infection. Inducing several public TCR clonotypes in CD8^+^ T cell populations via vaccination that are maintained after infection may, therefore, be efficacious in reducing acute and chronic phase viral loads.

## Supporting Information

Figure S1 **Viral load and GagCM9-specific CD8^+^ T cell tetramer staining for each animal.** (a–g) Plasma viral RNA content was measured by quantitative PCR from weeks 0 to 48 post-infection for all vaccinated animals, black diamonds and connecting line. The percentage of CD3^+^CD8^+^ GagCM9-specific T cells were enumerated using GagCM9-specific tetramers at each indicated timepoint, red triangles and connecting line. (h, i) Plasma viral RNA content (black diamonds and connecting line) and the percentage of CD3^+^CD8^+^ GagCM9-specific T cells (blue diamonds and connecting line) were calculated at each timepoint from the two mock-vaccinated, SIVsmE660-infected control animals. (j) The group mean viral load (black diamonds and connecting line) and GagCM9-specific CD8^+^ T cell tetramer percentage (red triangles and connecting line) of the seven vaccinated, SIVsmE660-infected animals were calculated.
(TIF)

Figure S2 **Pyrosequencing revealed less than 25% of viral populations exhibit GagCM9 escape in six of the seven animals.** Pyrosequencing was performed at weeks two, ten and twenty post-infection of the GagCM9 epitope and surrounding regions. We were unable to sequence virus from r02042 at week twenty and r02049 at weeks ten and twenty due to low or undetectable viral loads. Only r01056 had escape within the GagCM9 epitope at one timepoint in greater than 25% of viral populations.
(TIF)

Figure S3 **Post-infection whole PBMC IFN-γ ELISPOT.** Week three (a) and ten (b) post-infection whole PBMC IFN-γ ELISPOT heat maps indicate responses to peptide pools detected at each timepoint. Green squares indicate 50–200 spot forming cells (SFCs)/million PBMC, blue squares indicate 201–500 SFCs/million PBMC, yellow squares indicate 501–1000 SFCs/million PBMC, orange squares indicate 1001–2000 SFCs/million PBMC, and red squares indicate greater than 2001 SFCs/million PBMC. T: Tat; R: Rev; V: Vpr; X: Vpx; *: GagCM9; ∧: GagQI9. GagCM9 was also located in the Gag E peptide pool.
(TIF)
