# Supplementary figures and images for: GagCM9-Specific CD8+ T Cells Expressing Limited Public TCR Clonotypes Do Not Suppress SIV Replication In Vivo
Source: PLoS One. 2011 Aug 26;6(8):e23515. doi: 10.1371/journal.pone.0023515 (PMC3162554; doi:10.1371/journal.pone.0023515)

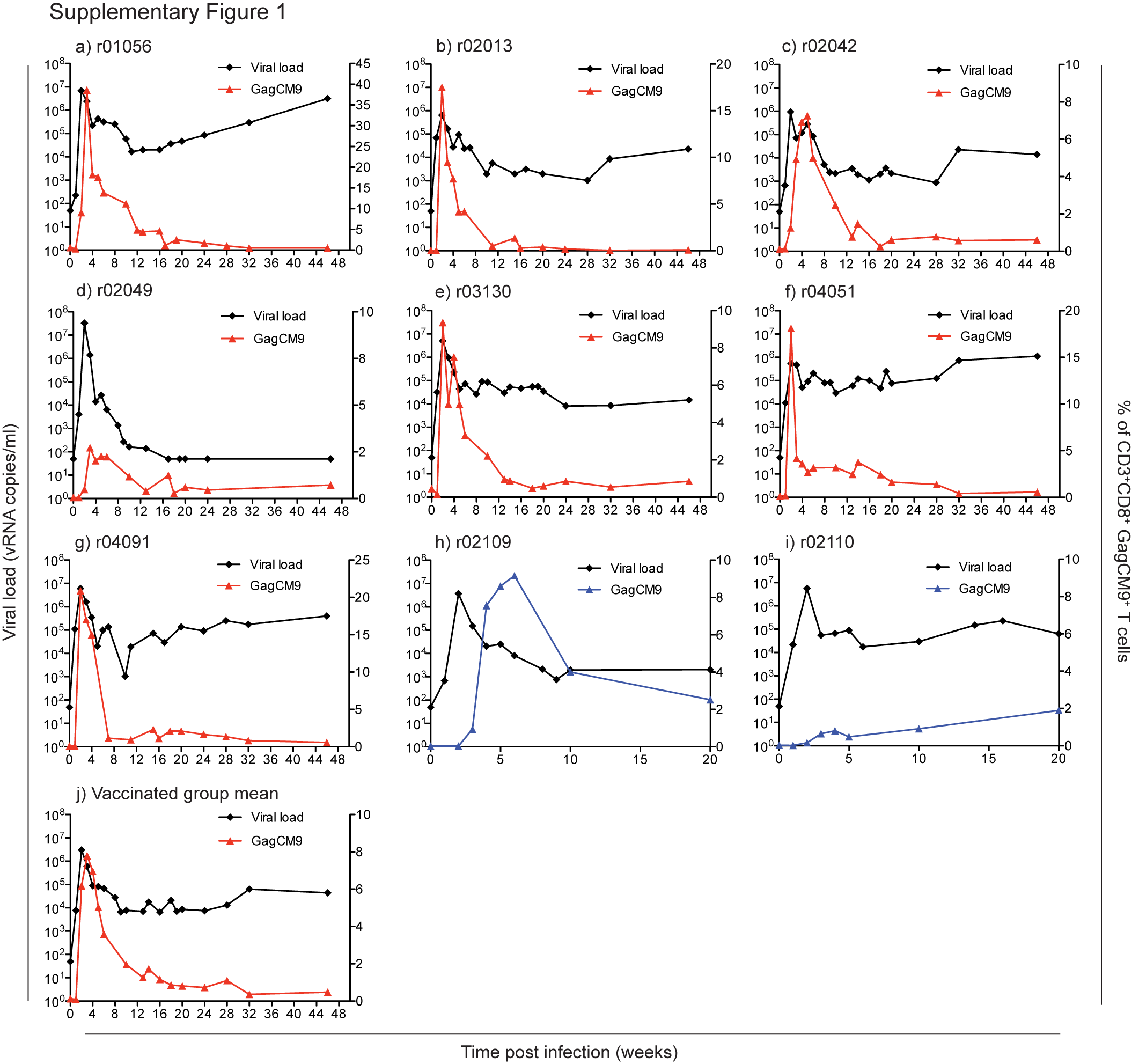

Supplement: Figure S1 — Viral load and GagCM9-specific CD8+ T cell tetramer staining for each animal. (a–g) Plasma viral RNA content was measured by quantitative PCR from weeks 0 to 48 post-infection for all vaccinated animals, black diamonds and connecting line. The percentage of CD3+CD8+ GagCM9-specific T cells were enumerated using GagCM9-specific tetramers at each indicated timepoint, red triangles and connecting line. (h, i) Plasma viral RNA content (black diamonds and connecting line) and the percentage of CD3+CD8+ GagCM9-specific T cells (blue diamonds and connecting line) were calculated at each timepoint from the two mock-vaccinated, SIVsmE660-infected control animals. (j) The group mean viral load (black diamonds and connecting line) and GagCM9-specific CD8+ T cell tetramer percentage (red triangles and connecting line) of the seven vaccinated, SIVsmE660-infected animals were calculated. (TIF) [file pone.0023515.s001.tif]

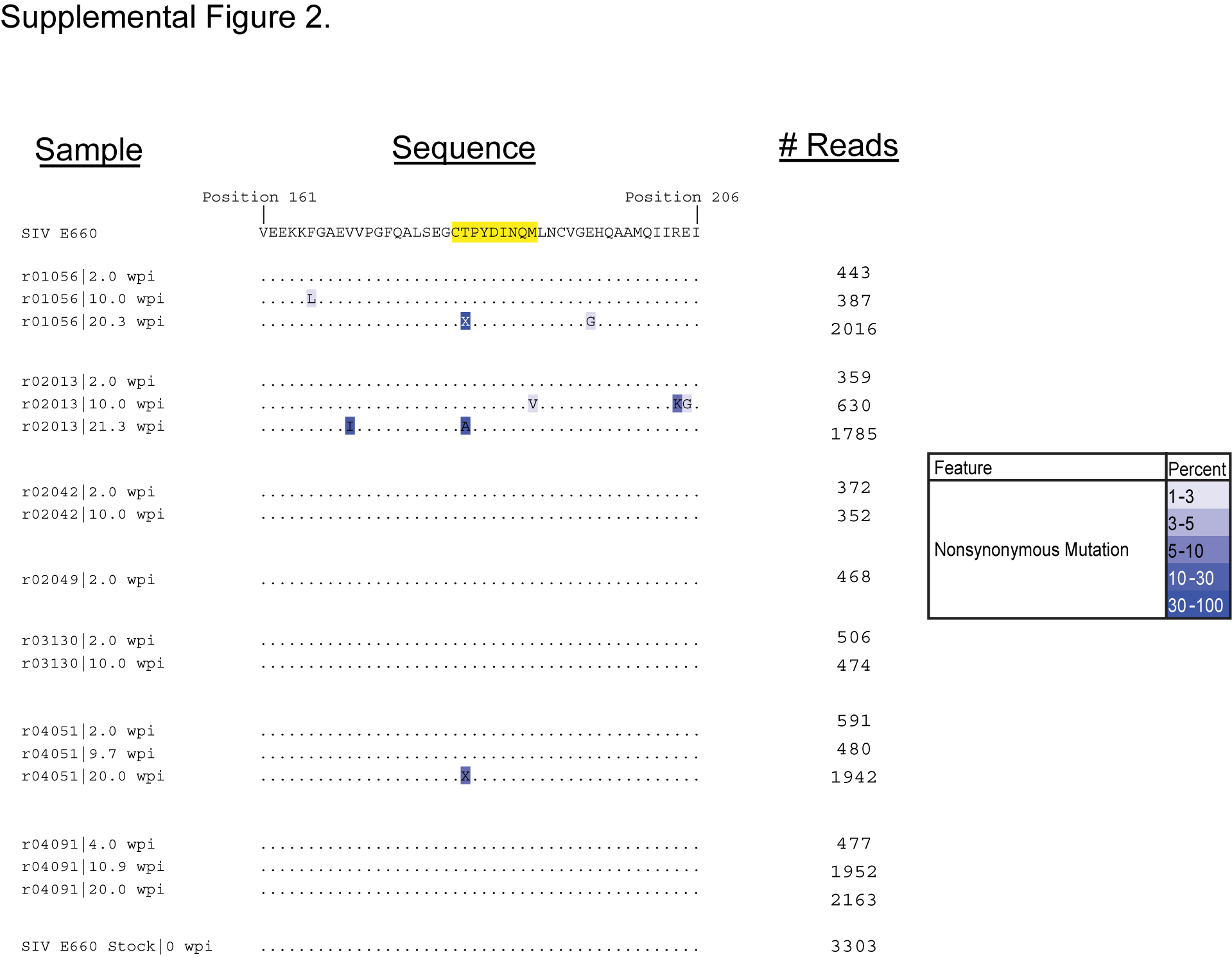

Supplement: Figure S2 — Pyrosequencing revealed less than 25% of viral populations exhibit GagCM9 escape in six of the seven animals. Pyrosequencing was performed at weeks two, ten and twenty post-infection of the GagCM9 epitope and surrounding regions. We were unable to sequence virus from r02042 at week twenty and r02049 at weeks ten and twenty due to low or undetectable viral loads. Only r01056 had escape within the GagCM9 epitope at one timepoint in greater than 25% of viral populations. (TIF) [file pone.0023515.s002.tif]

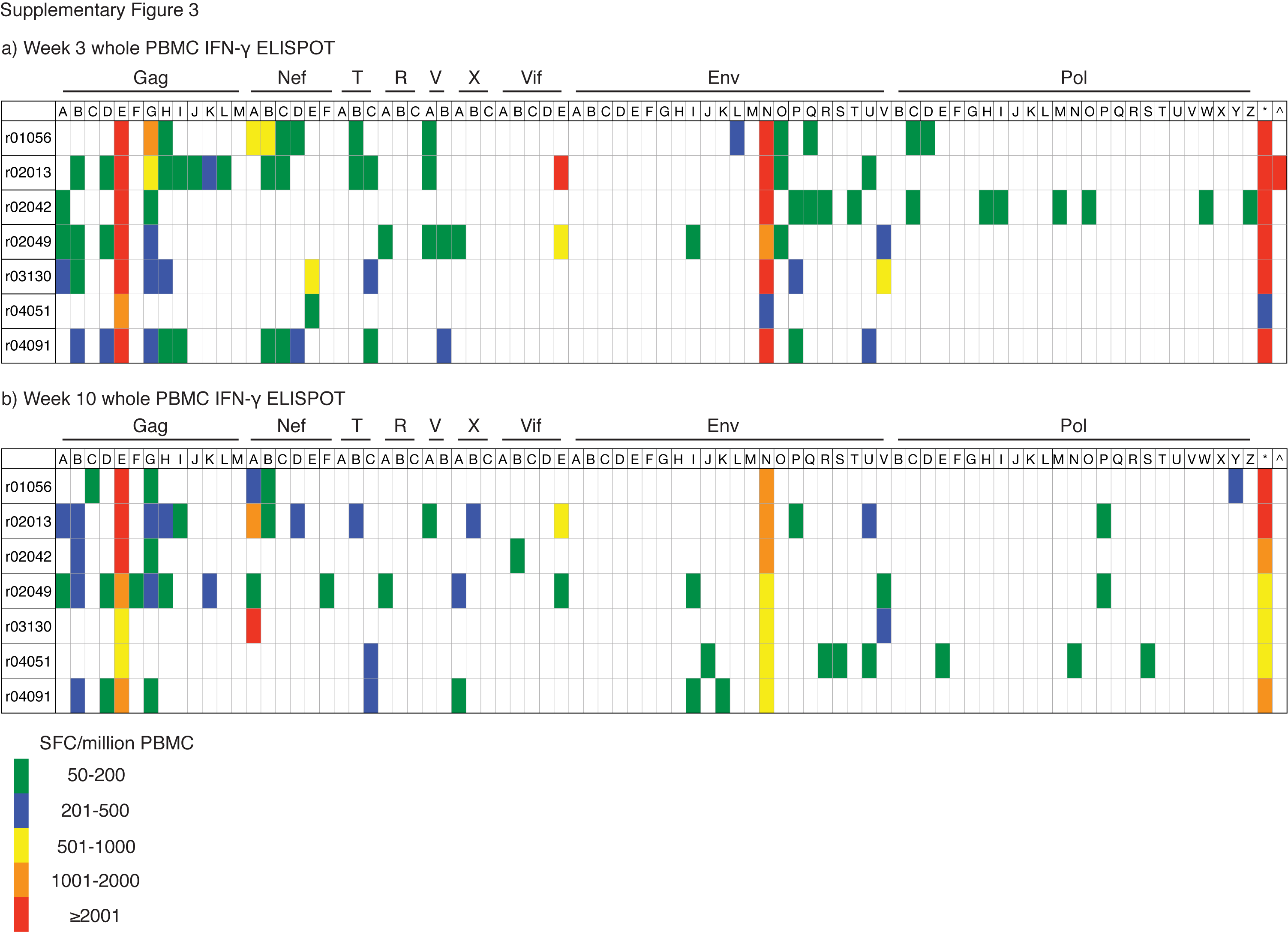

Supplement: Figure S3 — Post-infection whole PBMC IFN-γ ELISPOT. Week three (a) and ten (b) post-infection whole PBMC IFN-γ ELISPOT heat maps indicate responses to peptide pools detected at each timepoint. Green squares indicate 50–200 spot forming cells (SFCs)/million PBMC, blue squares indicate 201–500 SFCs/million PBMC, yellow squares indicate 501–1000 SFCs/million PBMC, orange squares indicate 1001–2000 SFCs/million PBMC, and red squares indicate greater than 2001 SFCs/million PBMC. T: Tat; R: Rev; V: Vpr; X: Vpx; *: GagCM9; ∧: GagQI9. GagCM9 was also located in the Gag E peptide pool. (TIF) [file pone.0023515.s003.tif]
